# Targeting Methylglyoxal in Diabetic Kidney Disease Using the Mitochondria-Targeted Compound MitoGamide

**DOI:** 10.3390/nu13051457

**Published:** 2021-04-25

**Authors:** Sih Min Tan, Runa S. J. Lindblom, Mark Ziemann, Adrienne Laskowski, Cesare Granata, Matthew Snelson, Vicki Thallas-Bonke, Assam El-Osta, Carlos D. Baeza-Garza, Stuart T. Caldwell, Richard C. Hartley, Thomas Krieg, Mark E. Cooper, Michael P. Murphy, Melinda T. Coughlan

**Affiliations:** 1 Department of Diabetes, Central Clinical, School, Alfred Medical Research and Education Precinct, Monash University, Melbourne, VIC 3004, Australia; sihmin.tan@monash.edu (S.M.T.); runa.lindblom2@monash.edu (R.S.J.L.); m.ziemann@deakin.edu.au (M.Z.); Adrienne.laskowski@monash.edu (A.L.); Cesare.granata@monash.edu (C.G.); matthew.snelson@monash.edu (M.S.); v.bonke@alfred.org.au (V.T.-B.); sam.el-osta@monash.edu (A.E.-O.); mark.cooper@monash.edu (M.E.C.); 2 Institute for Health and Sport, Victoria University, Melbourne, VIC 3011, Australia; 3 WestCHEM School of Chemistry, University of Glasgow, Glasgow G12 18QQ, UK; aquiles7389@hotmail.com (C.D.B.-G.); stuart.caldwell@glasgow.ac.uk (S.T.C.); Richard.hartley@glasgow.ac.uk (R.C.H.); 4 Department of Medicine, Cambridge Biomedical Campus, University of Cambridge, Cambridge CB2 0XY, UK; tk382@medschl.cam.ac.uk (T.K.); mpm@mrc-mbu.cam.ac.uk (M.P.M.); 5 MRC Mitochondrial Biology Unit, Cambridge Biomedical Campus, University of Cambridge, Cambridge CB2 0XY, UK; 6 Baker Heart & Diabetes Institute, Melbourne, VIC 3004, Australia

**Keywords:** sugar-derived products, methylglyoxal, dicarbonyl, diabetes, kidney, mitochondria, MitoGamide

## Abstract

Diabetic kidney disease (DKD) remains the number one cause of end-stage renal disease in the western world. In experimental diabetes, mitochondrial dysfunction in the kidney precedes the development of DKD. Reactive 1,2-dicarbonyl compounds, such as methylglyoxal, are generated from sugars both endogenously during diabetes and exogenously during food processing. Methylglyoxal is thought to impair the mitochondrial function and may contribute to the pathogenesis of DKD. Here, we sought to target methylglyoxal within the mitochondria using MitoGamide, a mitochondria-targeted dicarbonyl scavenger, in an experimental model of diabetes. Male 6-week-old heterozygous Akita mice (C57BL/6-Ins2-Akita/J) or wildtype littermates were randomized to receive MitoGamide (10 mg/kg/day) or a vehicle by oral gavage for 16 weeks. MitoGamide did not alter the blood glucose control or body composition. Akita mice exhibited hallmarks of DKD including albuminuria, hyperfiltration, glomerulosclerosis, and renal fibrosis, however, after 16 weeks of treatment, MitoGamide did not substantially improve the renal phenotype. Complex-I-linked mitochondrial respiration was increased in the kidney of Akita mice which was unaffected by MitoGamide. Exploratory studies using transcriptomics identified that MitoGamide induced changes to olfactory signaling, immune system, respiratory electron transport, and post-translational protein modification pathways. These findings indicate that targeting methylglyoxal within the mitochondria using MitoGamide is not a valid therapeutic approach for DKD and that other mitochondrial targets or processes upstream should be the focus of therapy.

## 1. Introduction

Chronic kidney disease (CKD) impacts more than 50 million individuals globally [1] and is a key risk factor for cardiovascular disease and all-cause mortality [2]. Up to 30% of the burden of CKD is a result of diabetes [3] and the progression to end stage renal failure in patients with diabetic kidney disease (DKD) is only slowed by current clinical therapies which attempt to target hyperglycemia and hypertension [4]. Sodium glucose cotransporter 2 (SGLT2) inhibitors are a new class of oral anti-hyperglycemic medications that are approved and indicated for type 2 diabetes and have shown promising renoprotective effects independent of their glucose lowering actions [5]. However, their use in type 1 diabetes is limited, mainly due to complications such as diabetic ketoacidosis as the main safety concern [6]. Therefore, there is a critical need to identify pathogenic factors responsible for the onset and progression of DKD especially in type 1 diabetes in order to develop new therapeutic targets.

The kidney is highly metabolically active, with the proximal tubules generating large quantities of adenosine triphosphate (ATP) via oxidative phosphorylation (OXPHOS), in order to drive active reabsorption of important macromolecules [7]. A large body of evidence indicates that mitochondrial dysfunction plays a central role in the development of DKD [8,9,10,11,12,13,14,15]. Some of the key features of disrupted mitochondrial homeostasis include mitochondrial ATP depletion, uncoupling, and fragmentation [16,17,18]. In addition, changes in the regulation of mitochondrial dynamics and bioenergetics are present prior to the development of early renal structural and biochemical lesions in diabetes [19].

Methylglyoxal and glyoxal are highly reactive dicarbonyl species derived from glucose and other reducing sugars, which accumulate in mitochondria during diabetes and form glycation adducts [15,20]. Methylglyoxal and glyoxal are also increased in foods during processing, thus are at increased levels in the typical Western diet [21]. Clinical studies have shown that plasma methylglyoxal levels are associated with a lower estimated glomerular filtration rate (eGFR) [22] and a higher urinary albumin-to-creatinine-ratio (ACR) [23] in diabetes. Furthermore, methylglyoxal-derived advanced glycation endproduct (AGE) levels, including methylglyoxal hydroimidazolone (MG-H1), correlate with structural kidney abnormalities in diabetes [24,25].

Methylglyoxal has also been linked to mitochondrial dysfunction. Mitochondrial proteins isolated from diabetic rat kidneys are a target of methylglyoxal-induced modifications and correlate with a defect in complex I-linked mitochondrial respiration [26]. In addition, decreasing methylglyoxal levels by overexpressing the enzyme glyoxalase-1 abolishes hyperglycemia-induced oxidative stress and renal injury in diabetic mice [27]. A newly developed mitochondria-targeted compound, MitoGamide, acts to sequester methylglyoxal and glyoxal in mitochondria [28], thereby inactivating these reactive products. MitoGamide is based on MitoG which was previously developed to assess the levels of methylglyoxal and glyoxal in the mitochondria [28]. However, MitoG is susceptible to oxidation and short lived. MitoGamide is a longer-lived analogue of MitoG and has been shown to be therapeutically relevant as it is protective in diabetic cardiomyopathy [29,30]. The current study aimed to ascertain if renoprotection could be achieved in the same mouse model using MitoGamide and explored the effects of this compound on restoring mitochondrial homeostasis and downstream signaling in the kidney.

## 2. Materials and Methods

### 2.1. Animals

All of the activities involving the use of animals for research were approved by the Alfred Research Alliance Animal Ethics Committee (Ethics number E/1502/2014/B) and were conducted according to guidelines of the National Health and Medical Research Council of Australia for animal experimentation. Ins2-Akita mice (C57BL/6J-Ins2Akita) and their wildtype (WT) littermates were purchased from the Jackson Laboratory and bred at the Alfred Research Alliance Animal Centre, Melbourne, Australia. Mice were maintained at the Alfred Research Alliance Animal Centre under a 12 h light/dark cycle. Animals were housed in groups of three mice per cage in a temperature-controlled environment and *ad libitum* access to food and water.

**Figure 1:**
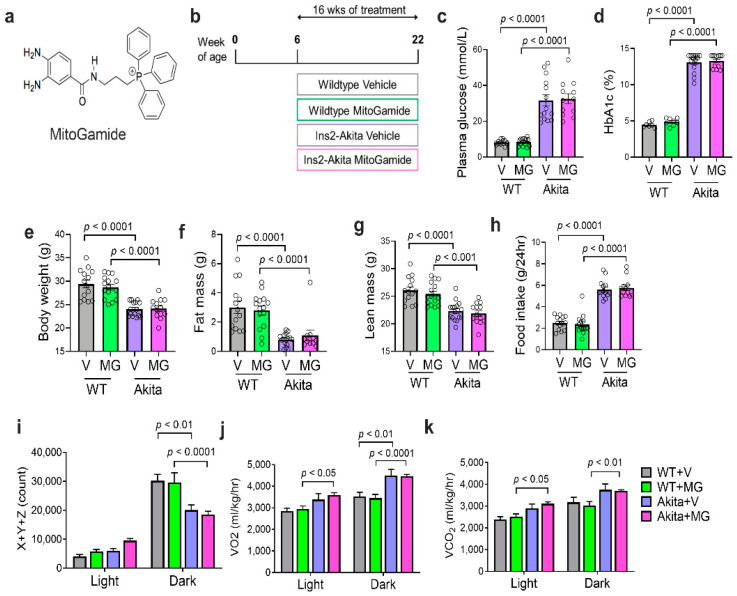
Characterization of diabetes, body composition, and physical activity of Akita mice following the MitoGamide treatment. (**a**) The chemical structure of MitoGamide; (**b**) schematic of experimental protocol. Plasma glucose (**c**), HbA_1c_, (**d**) and body weight (**e**) measured after 16 weeks of MitoGamide treatment. Body composition was measured by EchoMRI, including fat (**f**) and lean mass (**g**). Total food intake over a 24-h period (**h**). Physical activity as assessed by beam breaks in X + Y + Z planes over 24 h (**i**). Whole body oxygen consumption (**j**) and carbon dioxide production (**k**) were measured using the CLAMS metabolic chamber. Data are presented as mean ± SEM, *n* = 7–15 mice per group. Clear dots show the individual data points. Two-way ANOVA followed by Tukey’s post hoc test.

MitoGamide (Figure 1a) was synthesized as previously described [30]. At 6 weeks of age, male mice of both genotypes were randomly assigned to receive either vehicle (10% ethanol in water) or MitoGamide (10 mg/kg) by daily oral gavage (Figure 1b). Blood glucose (ACCU-CHEK glucometer, Roche) and body weights were recorded on a weekly basis as part of animal monitoring. Mice were placed individually into metabolic cages (Iffa Credo, L’Arbresele, France) and urine was collected for 24 h at mid-point (12 weeks of age) and end-point (21 weeks of age). At 17 weeks of age (11 weeks after the treatment), the body composition was determined using an EchoMRI (EchoMRI™, Houston, TX, USA) and mice were placed into individual Comprehensive Laboratory Animal Monitoring System (CLAMS, Columbus Instruments, Columbus, OH, USA) chambers to determine the physical activity and whole body respiration by indirect calorimetry, as previously described in full [31]. After 16 weeks of treatment, animals were euthanased by sodium pentobarbital (80 mg/kg i.p.) and the kidneys were rapidly excised, dissected, and prepared for mitochondria isolation (for real-time mitochondrial respiration studies, see below), snap-frozen or placed in a 10% neutral buffered formalin (*v/v*) for fixation before paraffin embedding. Glycated haemoglobin (HbA_1c_) was measured at the end of the study using the Cobas B 101 system (Roche Diagnostics Corporation, Indianapolis, IN, USA). Plasma glucose was measured using a glucose colorimetric assay kit (Cay·man, Ann Arbor, MI, USA).

### 2.2. Tissue Distribution of MitoGamide In Vivo

MitoGamide (10 mg/kg) was given to mice (*n* = 3) by oral gavage, then its contents in the heart, liver, and kidney were measured by LC-MS/MS, as previously described [30].

### 2.3. Renal Function and Morphometry

Mouse specific enzyme-linked immunosorbent assays (ELISA) were used to measure urinary albumin excretion (Bethyl Laboratories, Montgomery, TX, USA), urinary kidney injury molecule-1 (KIM-1, USCN Life Sciences, Wuhan, China), and serum cystatin C (BioVendor, Mordice, Czech Republic) as per the manufacturer’s specifications. The glomerulosclerotic index (GSI) was assessed in periodic acid Schiff (PAS)-stained sections, as previously described [32]. In brief, PAS-stained kidney sections were graded on a scale of 1–4: 1 = up to 25% sclerotic area, 2 = 25–50% sclerotic area, 3 = 50–70% sclerotic area, and 4 = more than 75% sclerotic area. GSI is then calculated as GSI = [(1 × N1) + (2 × N2) + (3 × N3) + (4 × N4)]/(N0 + N1 + N2 + N3 + N4), where nx is the number of glomeruli in each grade of glomerulosclerosis.

The immunohistochemistry for collagen IV and fibronectin were performed on paraffin-embedded kidney sections, as previously described [32] using a goat polyclonal anti-collagen IV antibody (Southern Biotech, Birmingham, AL, USA) and a rabbit polyclonal anti-fibronectin antibody (Abcam, Cambridge, UK), respectively. Tubulointerstitial fibrosis in kidney sections was examined using picrosirius red staining, as previously described [32]. Quantitation of immunohistochemistry (brown staining) and tubulointerstitial fibrosis (red staining) was performed by computer-aided densitometry (Image-Pro Plus 6.0, software v6.0, Rockville, MD, USA). Quantitation of renal morphology was performed in a blinded manner with the investigator unaware of the treatment groups of each kidney section.

### 2.4. Mitochondrial Oxygen Consumption

Mitochondria was isolated from freshly collected renal cortex [33]. Isolated mitochondria were used to measure real-time respiration, as previously described [33]. In brief, mitochondria were loaded into a XFe96well Seahorse Bioanalyzer plate (Seahorse Bioscience, Agilent, Santa Clara, CA, USA) with a minimum of five replicate wells per mouse. For complex I respiration, 0.4 μg of mitochondria was loaded per well with 10 mM glutamate and 10 mM malate. Basal respiration with substrates was measured twice for 3 min each, followed by subsequent injections of (A) 0.5 mM ADP (State 3o), (B) 2.5 μg/μL oligomycin (State 4), (C) 1 μM carbonyl cyanide trifluoromethoxy phenylhydrazone (FCCP) (State 3u), and (D) 4 μM antimycin-A. Data were analyzed per individual mouse with each injection point assessed for a successful response via pre-defined criteria [33]. Basal respiration (OCR before first injection minus OCR after antimycin A injection), ATP-linked respiration (OCR after ADP injection minus basal OCR), proton leak respiration (OCR after oligomycin injection minus OCR after antimycin A injection), maximal respiration (OCR after FCCP injection minus OCR after antimycin A injection), and spare respiratory capacity (OCR after FCCP injection minus basal OCR) were then calculated.

### 2.5. Mitochondrial Hydrogen Peroxide Production

Hydrogen peroxide production was measured in isolated mitochondria using the Amplex Red reagent, as previously described [34]. To assess complex I-stimulated hydrogen peroxide production, 10 mM glutamate and 10 mM malate were added, for complex II, 10 mM succinate and 5 μM rotenone (a complex I inhibitor) were added.

### 2.6. Western Immunoblotting

Renal cortices were homogenized using a Next Advance Bullet Blender 24 (Averill Park, NY, USA) at 4 °C, with 1.00 and 2.00 mm beads at speed 8 for 4 min in a RIPA extraction buffer (50 mM Tris-HCl, 150 mM NaCl, 1% Triton X-100, 0.5% sodium deoxycholate, 0.1% SDS) at pH 7.4, containing a protease/phosphatase inhibitor cocktail (#5872, Cell Signaling Technology, Danvers, MA, USA). The protein content was determined using a BCA Protein Assay Kit (Pierce-Thermo Fisher Scientific, Melbourne, Australia). Equal amounts of the protein were separated on 4–20% Mini-Protean TGX Stain-Free gels (Bio-Rad Laboratories, Gladesville, NSW, Australia). Gels were then transferred to a polyvinylidene fluoride (PVDF) membrane on Turbo-Transfer (Bio-Rad laboratories, Gladesville, NSW, Australia). Membranes were blocked in 5% skim milk-Tris buffered saline, 0.1% Tween-20 (TBS-T) for 1 h before blotting with a MG-H1 antibody (hm5017; Hycultec GmbH, Beutelsbach, Germany) overnight at 4 °C. After washing and incubating with an HRP-conjugated polyclonal anti-mouse secondary antibody (P0260, Agilent Technologies, Mulgrave, VIC, Australia), membranes were visualized using a ChemiDoc™ Touch Imaging System (Bio-Rad laboratories, Gladesville, NSW, Australia). Samples were normalized by whole-lane stain free quantification to verify equal loading between lanes.

### 2.7. RNA Sequencing and Analysis

Approximately 200 ng of total RNA underwent RNA depletion using the NEBNext rRNA Depletion Kit followed by library construction using an NEBNext Ultra II Directional RNA Library Prep Kit for Illumina (both from New England BioLabs Inc., Ipswich, MA, USA). Barcoded libraries underwent Illumina 100 cycle single-read sequencing at the Australian Genome Research Facility, Melbourne, using HiSeq v4 reagents. Reads underwent trimming in Skewer v0.2.2 [35] in order to remove bases with a quality less than Phred 20. Reads were then mapped to Gencode mouse v25 transcripts with kallisto v0.46.1 [36]. Tabular data were read into R v4.0.2 for downstream analysis. Transcript level counts were then aggregated to gene level counts. For each contrast, genes with an average of fewer than 10 counts were excluded. The resulting count matrix underwent differential analysis with DESeq2 v1.28.1 [37]. Mouse gene names were mapped to humans using data from Ensembl Biomart v101 [38]. The enrichment analysis was performed using mitch v1.0.8 [39] with gene sets from Reactome [40] (obtained 8 October 2020). Additionally, gene sets related to diabetes were obtained from MsigDB v7.2 [41]. Genes and gene sets with false discovery rate adjusted *p*-values (FDR) < 0.05 were considered statistically significant.

### 2.8. Statistical Analysis

Data were analyzed with the GraphPad Prism 7.01 statistical software package. All data, except RNASeq data (see above), are presented as the mean ± standard error of the mean (SEM). A two-way analysis of variance (ANOVA) was used to detect the main effects for genotype (WT vs. Akita) and treatment (Vehicle vs. MitoGamide), followed by Tukey’s post hoc test to analyze the differences between individual experiment groups. A *p* < 0.05 was considered as statistically significant.

## 3. Results

### 3.1. Characterization of Diabetes, Body Composition, Physical Activity, and Respiration

MitoGamide was detected in the heart, kidney, and liver in mice 1 h after oral gavage (Figure S1). In this study, at 22 weeks of age (see Figure 1b for experimental timeline), Akita mice exhibited a significant increase in plasma glucose (Figure 1c) and HbA_1c_ levels (Figure 1d). The body weight of Akita mice was significantly reduced when compared to their WT littermates (Figure 1e). The reduction in bodyweight of Akita mice coincided with a significant reduction in fat mass (Figure 1f) and lean body mass (Figure 1g). However, the total food intake was significantly increased in Akita mice when compared to WT mice (Figure 1h). The diabetes status and body composition of Akita mice were not altered by the MitoGamide treatment (Figure 1).

The daily physical activity of mice was assessed by ‘beam breaks’ in X + Y + Z directions in the CLAMS system over 24 h and divided across the light (inactive/sleep) and dark (active) cycles. No change in physical activity was observed across groups in the light cycle. However, Akita mice exhibited a significant reduction in physical activity compared to WT mice during the active phase, regardless of treatment (Figure 1i). The whole body respiration was analyzed via changes in oxygen consumption (VO_2_) and carbon dioxide production (VCO_2_). Akita mice had increased oxygen consumption (Figure 1j) and increased carbon dioxide production (Figure 1k) during the active cycle when compared to WT controls. This increase in energy expenditure partly explains why even with increased total food intake in a 24-h period, Akita mice still had reduced body weight when compared to the WT control. MitoGamide had no effect on the whole body respiration or eating behaviour in Akita mice.

### 3.2. MitoGamide Does Not Protect against Diabetes-Induced Renal Injury

**Figure 2:**
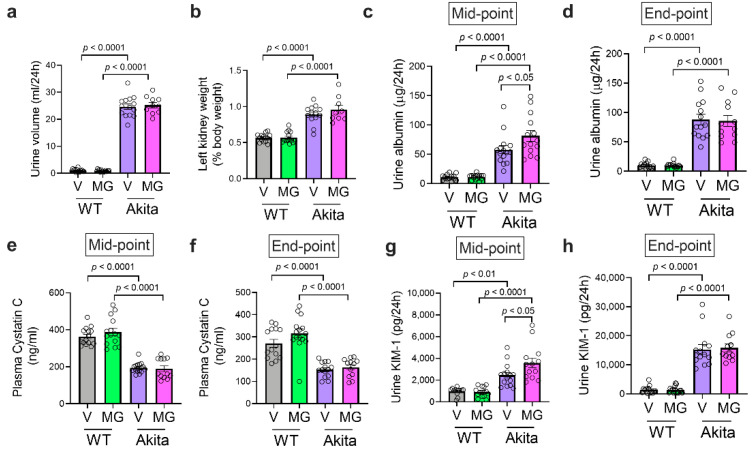
MitoGamide treatment does not attenuate diabetes-induced renal injury. (**a**) The 24-h urine volume was collected in metabolic cages. (**b**) The left kidney to body weight ratio after 16 weeks of MitoGamide treatment. Urinary albumin excretion at (**c**) mid-point and (**d**) end-point of the study. Plasma cystatin C at (**e**) mid-point and (**f**) end-point of the study. Urinary excretion of kidney injury molecule-1 (KIM-1) at (**g**) mid-point and (**h**) end-point of the study. Data are presented as mean ± SEM, *n* = 8–15 mice per group. Clear dots show individual data points. Two-way ANOVA followed by Tukey’s post hoc test.

As expected, diabetes was associated with an increase in urine volume (Figure 2a) and renal hypertrophy as shown as an increase in the left kidney to the body weight ratio (Figure 2b), both of which were not affected by the MitoGamide treatment. Urinary albumin was significantly increased in Akita mice, indicating a renal functional abnormality at both mid-point (Figure 2c) and end-point (Figure 2d) of the study. Interestingly, Akita mice treated with MitoGamide had a further increase in urinary albumin excretion at mid-point. However, there was no change in urinary albumin between vehicle and MitoGamide-treated Akita mice at the end-point of the study. Plasma cystatin C, a marker of glomerular filtration rate, was reduced in Akita mice at both mid-point (Figure 2e) and endpoint (Figure 2f), indicating that Akita mice exhibited hyperfiltration and this was not affected by the MitoGamide treatment. Urinary excretion of the tubular damage-associated marker, KIM-1, was increased in Akita mice and was not changed by the MitoGamide treatment (Figure 2g,h). Collectively, these results suggest that the treatment with MitoGamide does not impact on diabetes-induced renal injury in Akita mice.

### 3.3. MitoGamide Does Not Attenuate Renal Pathology in Akita Mice

**Figure 3:**
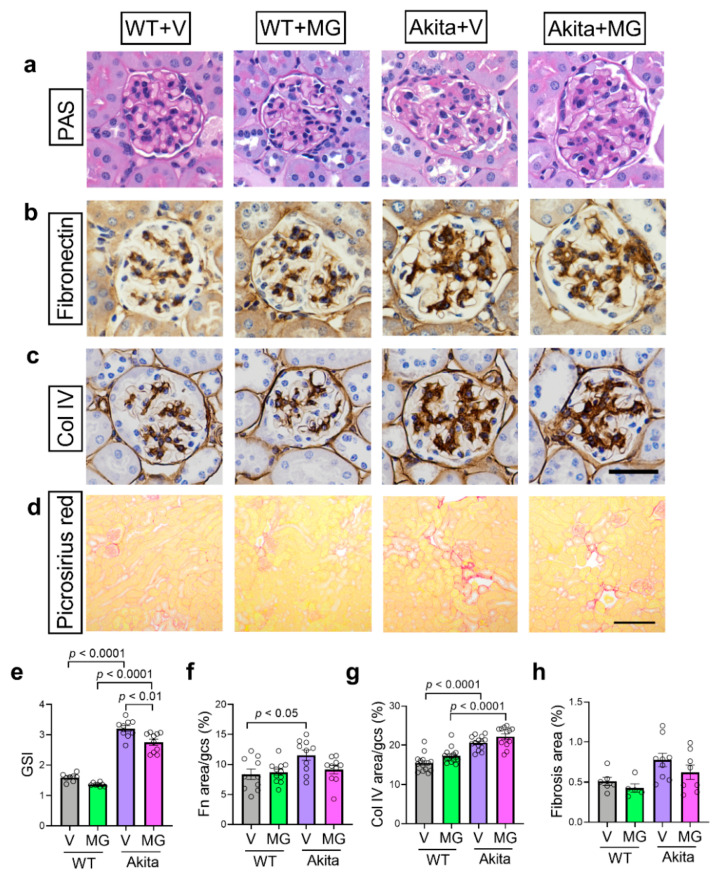
MitoGamide treatment does not attenuate diabetes-induced renal pathology. (**a**) Glomerulosclerotic index (GSI) was used to assess glomerulosclerosis in Akita mice after 16 weeks of MitoGamide treatment. Glomerular (**b**) fibronectin and (**c**) collagen IV were examined by immunohistochemistry. The scale bar is 50 μm. (**d**) Picrosirius red staining was used to assess tubulointerstitial fibrosis. The scale bar is 200 μm. Quantitation of (**e**) GSI, (**f**) fibronectin immunostaining, (**g**) collagen IV immunostaining, and (**h**) tubulointerstitial fibrosis area. Data are presented as mean ± SEM, *n* = 6–15 mice per group. Clear dots show individual data points. Two-way ANOVA followed by Tukey’s post hoc test.

Glomerulosclerosis in the kidney was assessed histologically. As expected, Akita mice had an increase in GSI when compared to WT controls (Figure 3a,e). After 16 weeks of MitoGamide treatment, there was a small, but significant, reduction of GSI in the Akita mice (Figure 3e). Although a similar trend was observed in glomerular fibronectin immunostaining, the reduction by MitoGamide in Akita mice was not significant (Figure 3b,f). No change was observed in the increase of glomerular collagen IV immunostaining in MitoGamide-treated Akita mice when compared to vehicle-treated Akita mice (Figure 3c,g). Picrosirius red staining was used to assess the degree of tubulointerstitial fibrosis. There was no significant difference in tubulointerstitial fibrosis across all groups, which is not surprising as the Akita mouse model bred onto the C57BL/6J background is known to not exhibit marked changes in the tubulointerstitium [42].

### 3.4. MitoGamide Does Not Inhibit the Formation of Renal Advanced Glycation Endproducts (AGEs) and Alter Mitochondrial Respiration

**Figure 4:**
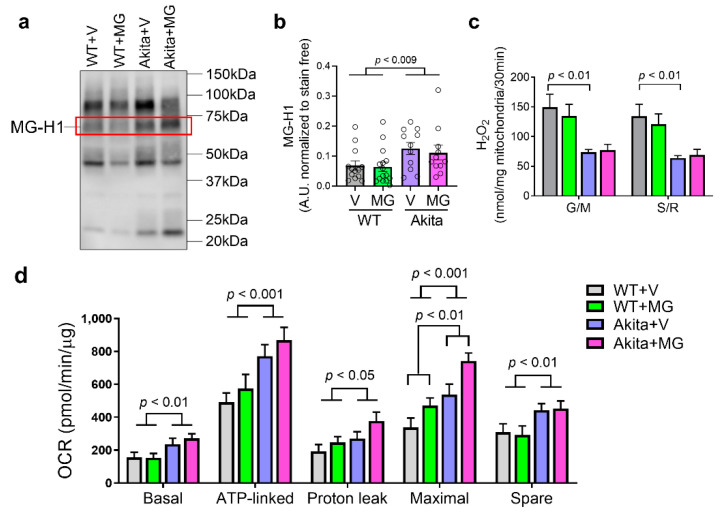
Akita mice exhibited changes in the kidney mitochondrial function with minor modulation by MitoGamide. (**a**) Methylglyoxal-derived hydroimidazolone 1 (MG-H1) examined by Western blotting (highlighted in red box) and (**b**) quantitation. (**c**) Hydrogen peroxide production in isolated mitochondria measured by Amplex red, in the presence of 10 mM glutamate and 10 mM malate (complex I) or 10 mM succinate and 5 μM rotenone (complex II). (**d**) Mitochondrial oxygen consumption rates (OCR) were measured by the Seahorse Bioanalyzer with glutamate + malate (G/M)-stimulated complex I respiration. Data are presented as mean ± SEM, *n* = 9–15 mice per group. Clear dots show individual data points. Two-way ANOVA followed by Tukey’s post hoc test.

Methylglyoxal-derived hydroimidazolone-1 (MG-H1) is the most common product in serum and tissues when methylglyoxal reacts with an arginine residue of a protein [20,43]. There was an overall increase in methylglyoxal-derived MG-H1 in the renal cortex in Akita mice (*p* < 0.0084) (Figure 4a,b), but this was unchanged with the MitoGamide treatment. The levels of H_2_O_2_ produced by isolated mitochondria from the kidney cortex were found to be significantly reduced in Akita mice with no difference between the vehicle and MitoGamide-treated mice (Figure 4c).

Renal cortical mitochondrial OCR were measured by the Seahorse Bioanalyzer using glutamate/malate to test the complex I-linked respiration. There was a significant increase in basal, ATP-linked, proton leak, maximal respiratory capacity, and spare OCR (Figure 4d) in cortical mitochondria from Akita mice. Interestingly, the maximal respiratory capacity of cortical mitochondria was significantly increased by MitoGamide in both WT and Akita mice (Figure 4d).

### 3.5. Characterization of the Kidney Transcriptome Using RNA Sequencing

**Figure 5:**
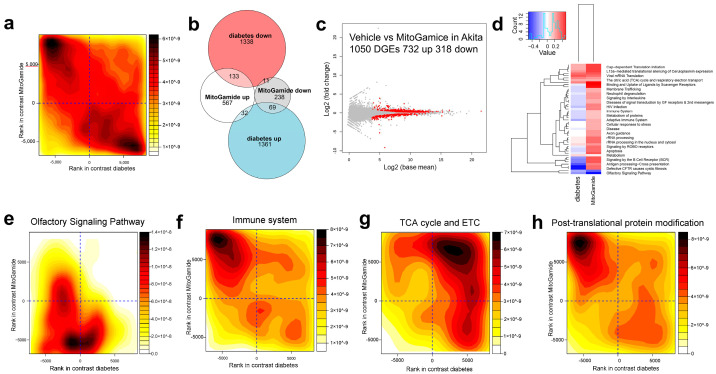
Characterization of transcriptome in kidney cortex of Akita mice following the MitoGamide treatment. (**a**) Rank-rank plot of gene expression differences due to diabetes and MitoGamide treatment. Genes were ranked by significance and the direction of the fold change. The top left corner shows that many genes which are downregulated in diabetes are restored by the MitoGamide treatment. The bottom right corner shows that genes which are upregulated by diabetes are attenuated by MitoGamide. (**b**) Venn diagram of MitoGamide attenuation of diabetes genes (FDR < 0.05). (**c**) MA plot of top differential genes in the Akita + vehicle and Akita + MitoGamide groups. (**d**) Gene set enrichment analysis of diabetes (Akita + vehicle) and MitoGamide (Akita + MitoGamide) groups, showing the top 25 Reactome pathways. Rank-rank density plots of differential genes in the (**e**) olfactory signaling pathway, (**f**) the immune system (**g**), the citric acid cycle (TCA) and respiratory electron transport (**h**), and post-translational protein modification pathways. The *x*-axis corresponds to the effect of diabetes (Akita mice) and the y-axis corresponds to the effect of MitoGamide.

Exploratory studies using transcriptomics in kidney cortex showed that gene expression changes in diabetes were largely attenuated by the MitoGamide treatment (Figure 5a). Approximately 5% of genes that were upregulated in diabetes were attenuated by the MitoGamide treatment and almost 9% of genes that were downregulated in diabetes were restored/upregulated by the MitoGamide treatment (Figure 5b). The differential gene expression (DGE) analysis identified 1050 DGEs, of which 732 were upregulated by the MitoGamide treatment in Akita mice and 318 were downregulated (Figure 5c). The reactome gene set analysis (Figure 5d) identified that the top 25 significantly altered pathways induced by the MitoGamide treatment are associated with the olfactory signaling pathway (Figure 5e), the immune system, including the immune system (Figure 5f), adaptive immune system, signaling by the B cell receptor (BCR), signaling by interleukins and neutrophil degranulation. In addition, gene sets associated with mitochondria such as the citric acid (TCA) cycle and respiratory electron transport were elevated in diabetes and exacerbated by the MitoGamide treatment (Figure 5g). Interestingly, gene sets associated with translational and post-translational of proteins, such as post-translational protein modification (Figure 5h), translational and metabolism of RNA were also affected by the MitoGamide treatment, which could partly explain why changes observed in the kidney transcriptome did not result in phenotypic changes [44].

## 4. Discussion

The key finding of this study is that the novel mitochondria-targeted compound, MitoGamide, does not offer renoprotection in the spontaneously diabetic Akita mouse model. After 16 weeks of treatment, MitoGamide exhibited no impact on the underlying diabetes phenotype, body composition or physical activity. The renal function and pathology were not affected by the MitoGamide treatment in Akita mice. Mitochondrial respiration was mildly affected by MitoGamide with a further increase in maximal capacity of the respiratory chain. RNA sequencing revealed significant changes in gene sets involved in the olfactory signaling pathway, the immune system and mitochondrial metabolism, and function in the kidney of Akita mice treated with MitoGamide. However, whether these transcriptomic changes translate to specific phenotypes requires further investigation. Importantly, these changes do not lead to major effects on diabetes-related renal functional or structural abnormalities.

Akita mice are a well characterized model of non-obese and hypo-insulinemic diabetes and exhibit more severe kidney dysfunction when compared to streptozotocin-induced diabetic mice of the same genetic background [45]. Unlike chemically-induced diabetic mice, Akita mice are not exposed to the confounding effects of enhanced oxidative stress caused by chemicals such as streptozotocin [46]. Furthermore, since the mitochondria appear to be a highly sensitive target for streptozotocin toxicity [47], genetic models of type 1 diabetes, such as the Akita mouse may be more suitable for studying diabetes-associated alterations in mitochondrial function and oxidative stress. We have previously shown that at 26 weeks of age, Akita mice display increased in albuminuria, glomerulosclerosis, and oxidative stress, which features a reminiscent of human DKD [48]. In this study, we observed a mild but significant increase in cortical mitochondrial basal, ATP-linked, proton leak, maximal respiratory, and spare capacity OCR in Akita mice, as well as changes in gene sets associated with the TCA cycle and respiratory electron transport. In a previous study by another group, kidney mitochondria isolated from 12 week old Akita mice showed induction of TCA cycle enzymes, however, mitochondrial respiration, ATP synthesis, and morphology were unaffected in the kidney [49]. In contrast, state 3 respiration, ATP synthesis, and mitochondrial cristae density were decreased in cardiac mitochondria and were accompanied by the coordinate repression of OXPHOS and peroxisome proliferator-activated receptor gamma coactivator 1-alpha (PGC-1α) transcripts, suggesting that changes in mitochondria in Akita mice are organ-specific and that there is an increased susceptibility of cardiac mitochondria to diabetes-induced dysfunction [49]. Thus, even though we have shown that MitoGamide offers cardioprotection in Akita mice using the same treatment regime as reported in this study [29], the lack of renoprotective effects of MitoGamide in the kidney could be partly due to Akita mice not displaying a significant mitochondrial dysfunction in the kidney.

Glycation is the non-enzymatic post-translational modification of proteins that is enhanced in diabetes, forming AGEs, and is associated with the development of diabetic complications [50]. The main precursors of AGEs are glucose and reactive dicarbonyls such as methylglyoxal and glyoxal [50]. Methylglyoxal and glyoxal react with lysine, arginine, and cysteine residues in proteins to form irreversible carbonyl adducts [30]. A previous study has shown that overexpression of a methylglyoxal detoxifying enzyme, glyoxalase-1 (Glo-1), reduces hyperglycemia-induced levels of carbonyl stress, AGEs and oxidative stress in streptozotocin-induced diabetic rats, demonstrating the link between glycation and oxidative stress in diabetes [51]. MitoGamide is a derivative of the mitochondrial probe, MitoG which was developed to assess the mitochondrial levels of methylglyoxal and glyoxal [28]. MitoGamide reacts with and sequesters glyoxal and methylglyoxal to form the inactive products methylquinoxaline amide (MQA) and quinoxaline amide (QA). Its enhanced stability is intended to enable MitoGamide to accumulate in mitochondria, sequester reactive 1,2-dicarbonyls and thereby decrease mitochondrial damage and ameliorate diabetic complications such as diabetic cardiomyopathy [30]. Surprisingly, although complications in Akita mice are primarily driven by high glucose, a previous study has shown that AGEs were not increased in the liver, kidney, plasma, and heart from Akita mice [30]. Although we observed a small but significant increase of MG-H1 in the kidney cortex, the lack of a reduction in MG-H1 accumulation in the kidneys of Akita mice with the MitoGamide treatment may be an explanation for why MitoGamide failed to confer renal benefits in this model.

While we did not observe significant changes to diabetes-induced renal phenotypes by the MitoGamide treatment in this study, the RNASeq analysis revealed several significant changes. Gene sets associated with the olfactory signaling pathway were further decreased in the diabetic kidney by MitoGamide. While originally thought to be restricted to the nose, key components of olfaction have been found to be expressed in the renal distal nephron and may play a role in the juxtaglomerular apparatus to modulate GFR, renin secretion, and blood pressure [52,53]. Furthermore, olfactory receptor, Olfr1393, is found to be expressed in the proximal tubular cells and is important for glucose handling and Sglt1 regulation, with Olfr1393 knockout mice exhibiting improved glucose tolerance that likely stems from urinary glucose wasting [54]. In this study, we found that gene sets associated with the olfactory signaling pathway were downregulated in diabetes and MitoGamide further reduced these transcripts. The implications of these changes require further investigation but are unlikely to be directly linked to renal injury in diabetes.

Furthermore, several immune-associated pathways were modulated by MitoGamide in the diabetic kidney. Methylglyoxal is an effective modifier of immune function, including modulation of dendritic cell function [55], causing multiple and varied immune-deficiencies and reducing the ability of immune cells to respond appropriately to stimuli [56]. However, renal cortical methylglyoxal levels were not changed by MitoGamide in this study, thus whether MitoGamide affects the immune response in this model is not known. We have shown that MitoGamide was able to largely restore the downregulation of gene sets associated with the immune system in this study by RNASeq. However, it should be noted that the Akita mouse model is not typically an inflammatory model and does not develop tubular injury and macrophage infiltration up to 5 months of age [42]. Thus, it is difficult to interpret how changes in gene sets associated with the immune function in Akita mice may translate to relevant phenotypes.

Type 2 diabetes is the more common form of diabetes and thus warrants further investigation. Recent findings from various trials of SGLT2 inhibitors including the renally dedicated CREDENCE (Canagliflozin and Renal Events in Diabetes With Established Nephropathy Clinical Evaluation) trial showed that SGLT2 inhibition reduced the risk of cardiovascular and renal events in type 2 diabetes regardless of HbA_1c_ [57], suggesting that the management of complications associated with type 2 diabetes continues to improve. However, the use of SGLT2 inhibitors in type 1 diabetes is still not generally recommended and may indeed be contraindicated due to the increased risk of ketoacidosis. Thus, novel therapies are urgently needed to reduce the burden of complications including renal disease associated with type 1 diabetes. Limitations of the current study include using a prophylactic regimen rather than a therapeutic approach where therapy is delayed until early signs of disease are evident, a common clinical scenario for treating and retarding diabetic complications. In addition, given the fact that liver has the highest tissue distribution of Mitogamide, future studies should examine the effects of MitoGamide on the liver. Lastly, the sample size in this study is relatively small. Another potential pitfall of the study is the potential occurrence of hyperglycemia induced hyperdiuresis and volume depletion. However, all mouse models of DKD display hyperglycaemia and renal hyperfiltration leading to excess urine output as a part of the pathophysiology of DKD.

## 5. Conclusions

Taken together, our data indicate that the Akita mouse model may not be a suitable model to study the renoprotective effects of MitoGamide. While showing some potential benefits of sequestering reactive dicarbonyls using MitoGamide through RNASeq, further work is required to fully elucidate the translational benefits of these results in other models of DKD, which display more prominent renal inflammation and potentially more severe renal mitochondrial abnormalities.

## Data Availability

Sequence data have been deposited in the National Center for Biotechnology Information Gene Expression Omnibus with accession number GSE159882.

## Supplementary Materials

The following are available online at https://www.mdpi.com/article/10.3390/nu13051457/s1. Figure S1: Tissue distribution of MitoGamide in mice.
