# Supplementary material for: Targeting Methylglyoxal in Diabetic Kidney Disease Using the Mitochondria-Targeted Compound MitoGamide
Source: Nutrients. 2021 Apr 25;13(5):1457. doi: 10.3390/nu13051457 (PMC8145135; doi:10.3390/nu13051457)
Supplement: Supplementary file 1 [file nutrients-13-01457-s001.zip › nutrients-1148885-supplementary.pdf]

Targeting Methylglyoxal in Diabetic Kidney Disease Using the Mitochondria-Targeted Compound MitoGamide

Supplementary Materials

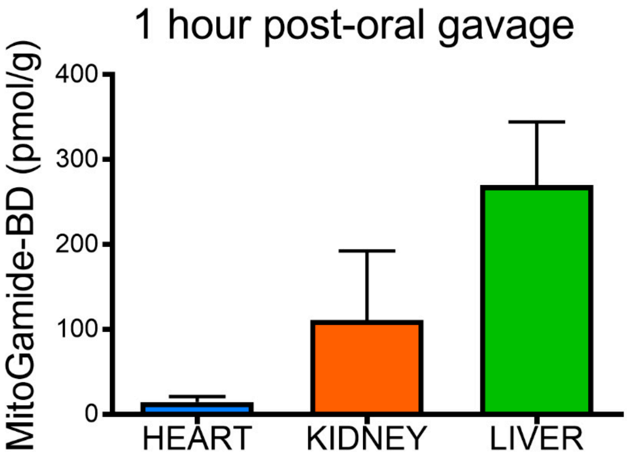

Figure S1. Tissue distribution of MitoGamide in mice. MitoGamide level was measured in heart, kidney and liver using LC-MS/MS after MitoGamide (10 mg/kg/body weight) was administered to mice by oral gavage.
